# Non-invasive measurement of accelerated gastrointestinal transit in pediatric patients using Contrast-enhanced Multispectral optoacoustic tomography

**DOI:** 10.1038/s44303-026-00169-4

**Published:** 2026-05-04

**Authors:** Luisa Caselitz, Merle Claßen, Adrian Buehler, Lars-Philip Paulus, Henriette Grieshaber-Bouyer Mandelbaum, Emmanuel Nedoschill, Joachim Wölfle, André Hoerning, Ferdinand Knieling, Adrian Philip Regensburger, Felix Wachter

**Affiliations:** Department of Pediatrics and Adolescent Medicine, University Hospital Erlangen, Erlangen, Germany

**Keywords:** Gastroenterology, Medical research

## Abstract

Functional intestinal disorders are common; however, current imaging modalities are limited. Contrast-enhanced Multispectral optoacoustic tomography (CE-MSOT) represents a novel, non-invasive, and radiation-free imaging technique that enables assessment of gastrointestinal transit time through the use of orally administered contrast agents. This could aid in symptom evaluation and diagnostic clarification in bowel disorders. A clinical pilot-study was conducted (Registry: ClinicalTrials.gov, TRN: NCT06617364, Registration date: 24 September 2024) involving 10 pediatric patients presenting with gastrointestinal complaints undergoing a lactose hydrogen breath test. CE-MSOT was performed following the oral administration of Indocyanine Green (ICG) as a contrast agent. In the terminal ileum and sigmoid colon multispectral optoacoustic tomography (MSOT) signals were spectrally unmixed to isolate ICG-specific signals. The appearance of ICG in these segments was used to estimate gastrointestinal transit time. ICG signals were detected in the terminal ileum as early as 13 min after oral administration (median: 80 min), and in the sigmoid colon as early as 41 min (median: 125 min).

## Introduction

Pediatric gastroenterology encompasses a broad spectrum of diseases that affect gastrointestinal function in children^1–3^. This includes well-established clinical entities such as inflammatory bowel disease (IBD), lactose intolerance, and celiac disease. Although functional abdominal pain disorders (FAP) are well known, too, the clinical assessment is challenging. FAP are common and impose a financial burden^4^ and significant deterioration in the quality of life^5,6^. A considerable number of pediatric patients present with persistent and unclear gastrointestinal symptoms—such as abdominal pain and diarrhea—yet remain undiagnosed despite extensive diagnostic evaluations^4,7,8^. This presents a significant clinical challenge, given the complex interplay between gastrointestinal symptoms and psychosocial factors in children, which can obscure the underlying pathophysiology^9–11^. These findings underscore the pursuit of a deeper understanding and objectification of unexplained gastrointestinal symptoms. Conventional diagnostic methods often fail to detect functional abnormalities in the absence of structural or biochemical changes^12,13^, leaving clinicians with limited tools to evaluate symptom severity and cause. Gastrointestinal transit time presents a logical target for functional assessment; however, current methods to measure it are either invasive, expose patients to radiation, or are not feasible in routine clinical practice^14,15^.

Multispectral optoacoustic tomography (MSOT) offers a promising, non-invasive solution to this diagnostic gap^16^. MSOT is an imaging modality that integrates optical and ultrasound technologies to visualize gastrointestinal tissue components and dynamic processes in real time^17^. Its utility has been demonstrated in adult^18^ and pediatric inflammatory bowel diseases^17^ to assess disease activity and various other clinical scenarios^19,20^. When combined with oral contrast agents, it has been shown to be able to record the intestinal transit time in healthy adults^16^. Especially in the pediatric setting, the measurement of gastrointestinal transit by contrast-enhanced MSOT (CE-MSOT) could serve as a valuable diagnostic tool to better characterize, understand, and contextualize individual gastrointestinal symptoms. To explore this potential, we conducted a pilot study in pediatric patients with heterogeneous gastrointestinal symptoms of unclear origin to evaluate the clinical feasibility of CE-MSOT for assessing gastrointestinal transit in children.

## Results

### Clinical trial and feasibility

From October 2023 to August 2024, a total of 10 patients were included in the study at the Department of Child- and Adolescent Medicine at the University Hospital Erlangen. Baseline characteristics of the study cohorts are provided in Table 1.

**Table 1:** Patient demographic

	mean ± SD
age [years]	10.9 ± 3.5
height [cm]	147.7 ± 23.3
weight [kg]	41.8 ± 19.0
BMI [kg/m²]	18.7 ± 3.9
sex F	6 (60%)
H2-breath-test positive	3 (30%)
symptoms during examination	4 (40%)
abdominal pain	8 (80%)
diarrhea	1 (10%)
obstipation	5 (50%)
diarrhea and obstipation	2 (20%)

Data in Table 1 characterizes the cohort. Values are numbers and percentage or mean ± standard deviation.

Patients were surveyed about their stool consistency before the examination and about their symptoms both before and during the examination.

*SD* Standard deviation, *BMI* body-mass-index.

**Fig. 1:**
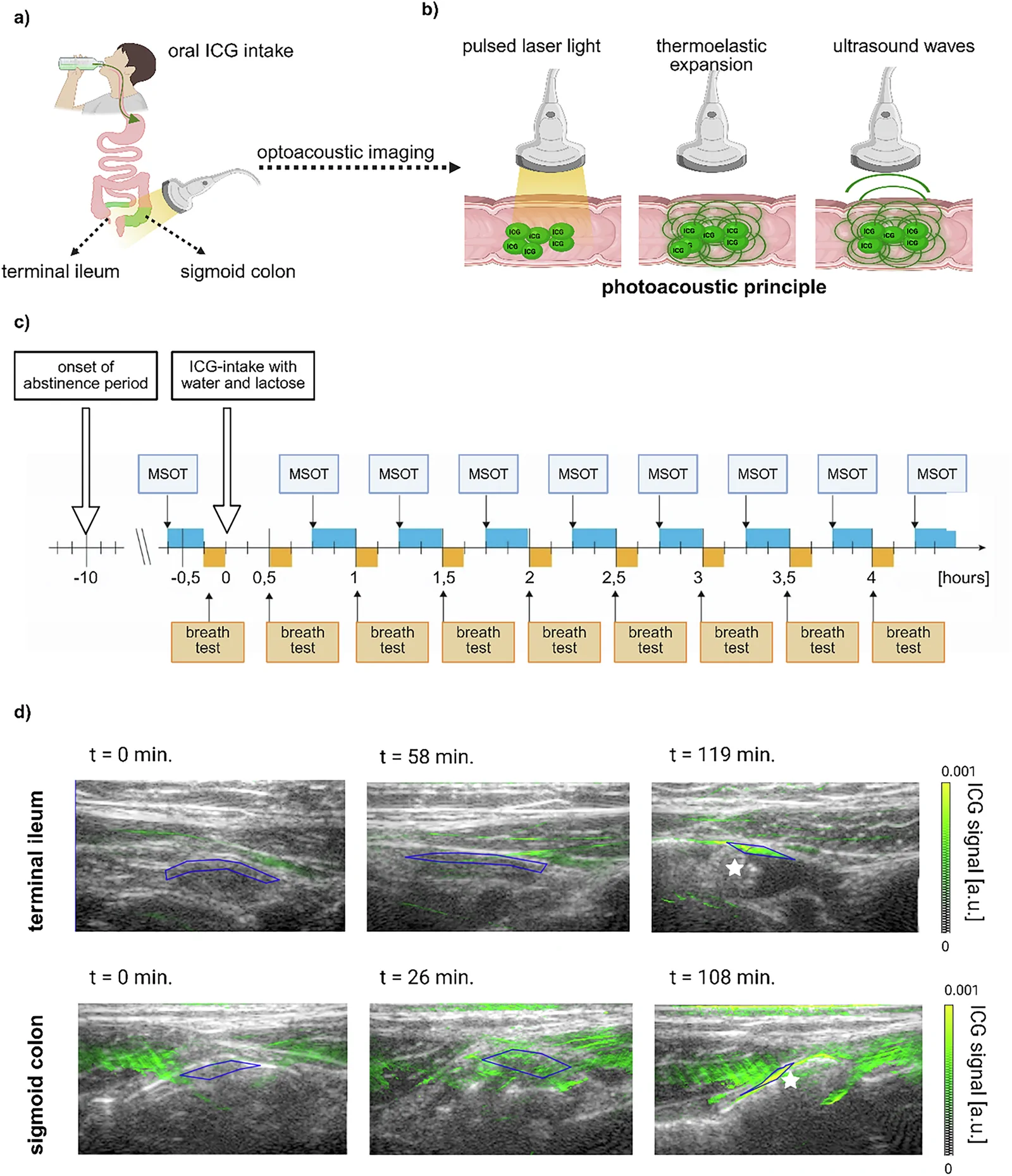
Study overview. **a** Multispectral optoacoustic imaging. Patients received a lactose and ICG-containing drink. Terminal ileum and sigmoid colon were assessed. **b** Photoacoustic principle. The handheld MSOT probe and generates pulsed laser light to induce thermoelastic expansion. The resulting optoacoustic spectra enable spectral unmixing and detection of exogenous chromophores such as ICG. **c** Examination protocol. Baseline measurements and ICG intake, were followed by repeated measurements (breath test and CE-MSOT) at 20–30-minute intervals. **d** CE-MSOT Imaging of a participant. Blue line shows the ROI as segmented by B-mode co-registration. Visual detection of ICG signals is indicated by asterisk.

The patients had a mean age of 10.9 ± 3.5 years, with the youngest being seven and the oldest 16 years old. Eight of the patients reported abdominal pain for at least the past four months, three reported symptoms for at least 4 years without a diagnosed cause. Other symptoms included nausea, diarrhea and obstipation. A positive hydrogen(H2) breath test was observed in three patients, however, only one of them experienced symptoms during the test. The CE-MSOT examination was successfully integrated into the clinical workflow without disruption of the H2 breath test measurements. Healthcare staff reported that the process was easy to implement and did not interfere with routine care. Importantly, no adverse reactions or safety concerns were observed in any participants throughout the study period. Already during the bedside examination, visual detection of ICG in the intestinal segments was feasible (Fig. 1).

**Fig. 2:**
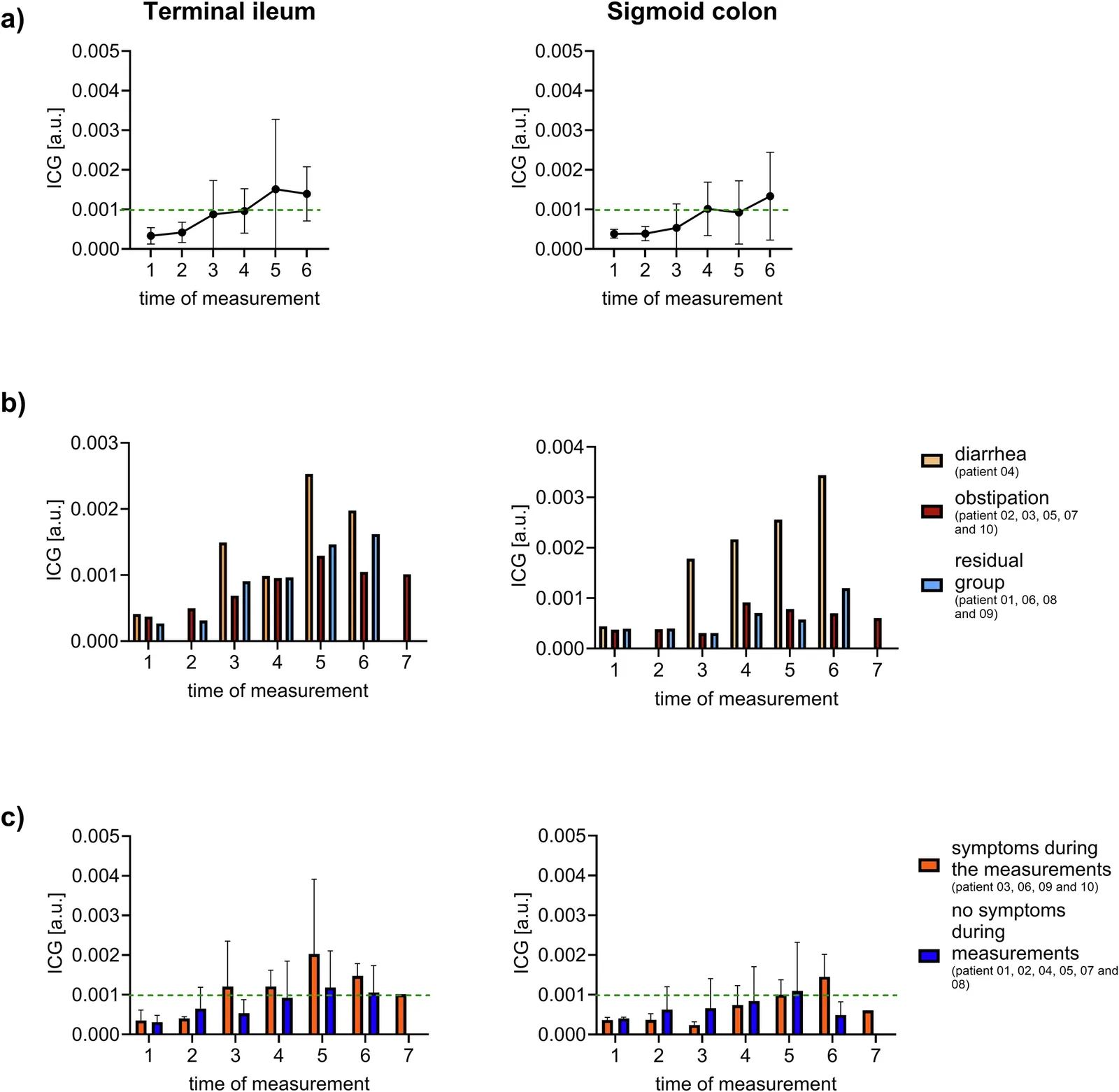
ICG level during the examination periods. **a** ICG signal across all patients. Mean values and standard deviation (SD) of the ICG signal at each time point in the terminal ileum and sigmoid colon. Time point 1 is the baseline measurement. The green line indicates the cut-off value. **b** ICG signal based on stool frequency and consistency in terminal ileum and sigmoid colon. Patients were categorized based on medical history into three groups: diarrhea, obstipation, and patients with normal stool frequency and consistency or with both diarrhea and obstipation. **c** ICG measurements based on clinical symptoms. ICG measurements (mean values and standard deviation of the ICG signal at each time point) in patients who did and did not report symptom onset during the examinations.

Furthermore, a quantitative cut-off point was implemented. The cut-off was determined by taking the highest baseline measurement prior to the oral administration of ICG and multiplying it by a factor of two, resulting in a threshold of 0.001 a.u., to create highest specificity (Fig. 2). Subsequently, we evaluated whether the measurements that were visually classified as positive also exceeded the defined cut-off value. This was confirmed in all cases. Likewise, all measurements in which ICG was not visually detectable remained below the quantitative threshold.

### Contrast-enhanced MSOT measured individual gastrointestinal transit times

**Fig. 3:**
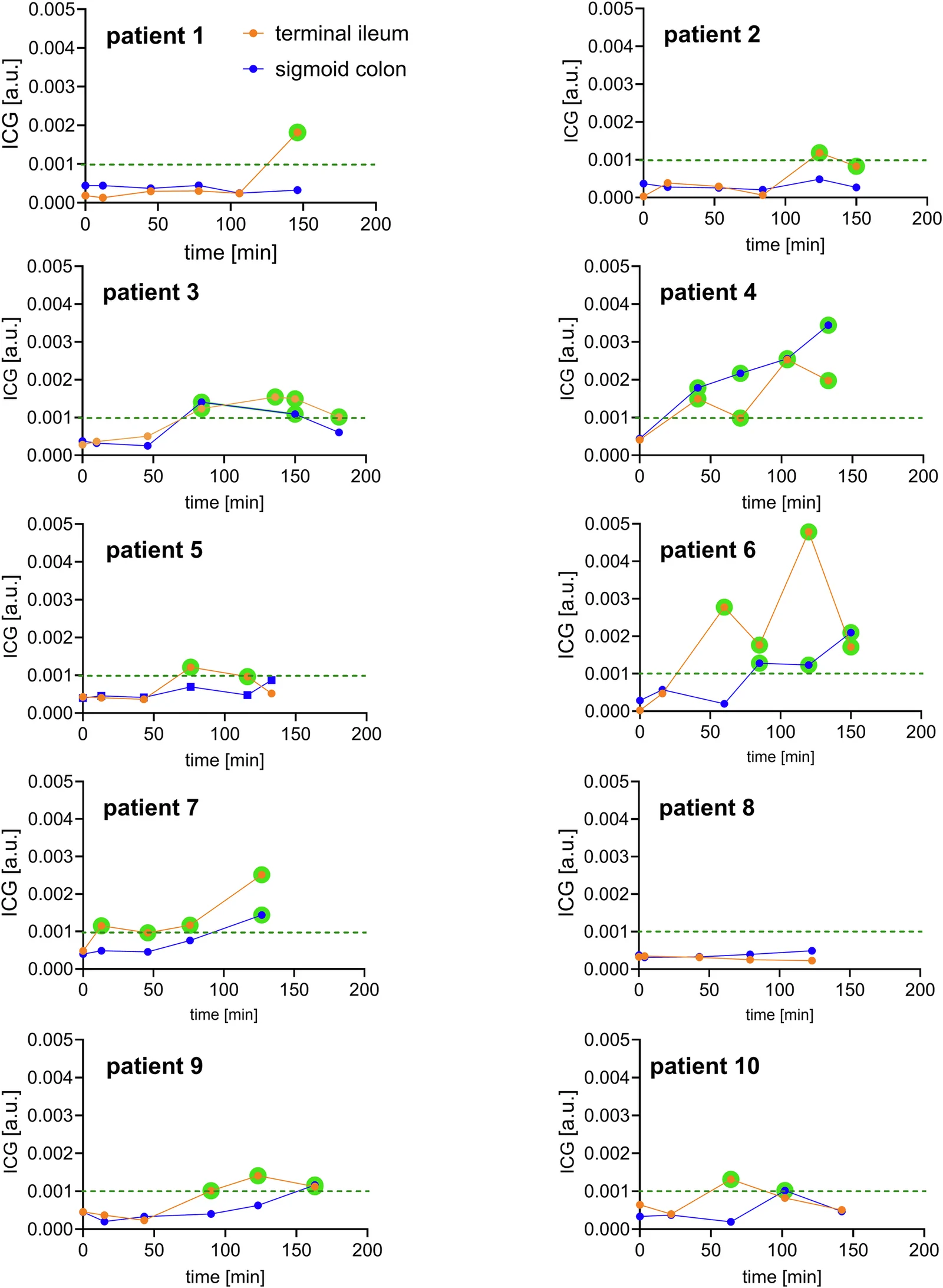
Overview of ICG signals for all study participants. Green circles highlight measurement points with visually detectable ICG signal. The green line serves as a cut-off value for ICG detection.

The median time required for transit to the terminal ileum was 80 min (range 13–146 min, interquartile range (IQR) 68 min). In one patient, no ICG was detected in the terminal ileum within the observation period (123 min), suggesting a longer transit time (Fig. 3). For the sigmoid colon, the median transit time was 125 min (range 41–163 min, SD 36.02 min). In four patients, no ICG signal was detected in the sigmoid colon during the individual study periods (sigmoid transit time of more than 123 to 150 min). In general, the measurements show a progressive increase in ICG signal intensity, with a peak observed in the terminal ileum at the fifth time point and in the sigmoid colon at the sixth and final time point (Fig. 2). In patients with constipation, one would typically assume a delayed transit. However, it is important to differentiate between transit through the small intestine and transit through the large intestine. For example, patients 2 and 5, both of whom reported symptoms of obstipation, showed a relatively rapid transit through the ileum (124 and 76 min). Nonetheless, a slower transit could be detected in the sigmoid colon (no signal during the respective measurement periods) (Fig. 3).

Green circles highlight measurement points with visually detectable ICG signal. The green line serves as a cut-off value for ICG detection.

This showed that the obstipation symptoms are not caused by reduced motility or delayed transit in the small intestine, but rather that the pathology is localized in the large intestine. Patient 4 reported the most severe symptoms, including diarrhea, occurring both during the day and at night. As early as 41 min after, an ICG signal was detected in both terminal ileum and the sigmoid colon, 84 min faster than the median sigmoid transit time and fastest of all measurements. These findings indicate a markedly accelerated gastrointestinal transit through both the small and large intestine, which is consistent with the patient´s pronounced clinical symptoms (Fig. 3).

### Abnormal contrast-enhanced MSOT finding in pediatric patients of different diseases

Although the patients were heterogeneous in many respects, certain commonalities were observed. Patients 3, 6, 9, and 10 reported symptoms during the course of the examinations. These symptoms primarily manifested as nonspecific abdominal discomfort and bloating, and notably, they developed only as the examination progressed. While these participants showed median transit times of 74 min to the terminal ileum and 94 min to the sigmoid colon, those without symptoms during the examination exhibited median transit times of 99.5 min to the terminal ileum and 130 min to the sigmoid colon, respectively. As illustrated in Fig. 2, the ICG signals in these patients showed an earlier and more pronounced increase. This was particularly evident in the terminal ileum, where the symptomatic group exceeded the defined cut-off point as early as the third measurement, whereas the asymptomatic group did so only after the fifth measurement. While notable differences in ICG intensity were also observed in the sigmoid colon—especially during the sixth measurement—these findings were less distinct than those in the terminal ileum. In conclusion, the onset of symptoms shortly after the start of the investigation appears to be associated with a more rapid transit and thus increased motility in the gastric or small intestinal segments. Notably, patients 3,6 and 9 did not exhibit a positive hydrogen breath test. However, they experienced symptoms following the ingestion of the lactose-containing beverage and demonstrated an unusually rapid intestinal transit. Patient 10 had a positive breath test, suggesting a causal relationship between the accelerated transit to the terminal ileum and lactose malabsorption. Another participant (patient 7) with a positive breath test was asymptomatic, however, he exhibited the fastest ileal transit time with a normal sigmoid transit time, indicating lactose malabsorption despite the absence of symptoms during the examination.

## Discussion

MSOT is an emerging imaging modality at the doorway of entering clinical routine^19^ that has been used to assess intestinal inflammation^21^, and to diagnose cardiovascular^22^ and oncologic diseases^23^. It is therefore necessary to carry out pilot studies in children to enable access to this novel technology and promote clinical use.

In this study, for the first time the feasibility and capability of CE-MSOT to assess gastrointestinal transit in pediatric patients was demonstrated. Previous studies have demonstrated the use of CE-MSOT for the assessment of gastrointestinal transit time in adults and preclinical models, only^16,19,24^.

Measuring pediatric patients with various abdominal complaints, we found some exceptionally short transit times for the ileum section of 80 min (range 13–146 min) and for the colon section of 125 min (range 41–163 min). The intestinal transit times observed in our study showed substantial deviations from the reference values reported for healthy pediatric populations. The median transit time to the terminal ileum reported in other studies using an electromagnetic 3D-Transit system in healthy children is 6.8 h (range 1.2–37.2 h), and to the sigmoid colon is 24.7 h (range 1.5-119.2 h)^25^. These deviations are a reflectance of the patients’ symptoms but might partially also be explained by fasting before the examination and the liquid intake of ICG.

Thus, in prior CE-MSOT studies^16,26^, transit times were more alike, even though the study participants were adults. For instance, increases of ICG signal in the terminal ileum in healthy adults were shown after 4.0–5.5 h^16^. In cystic fibrosis patients the ileum transit time has been shown to be accelerated to 2.0 h^26^.

For the assessment of gastrointestinal transit, several established diagnostic tools are available including wireless motility capsules (smart pills), scintigraphy and MRI. However, smart pills are relatively invasive and contraindicated in patients with certain pathologies such as gastrointestinal stenosis. Scintigraphy involves exposure to ionizing radiation and a single MRI acquisition may take up to 40 minutes. In contrast, CE-MSOT measurements can be completed in less than 5 minutes. Due to its non-invasive and user-friendly nature CE-MSOT imposes a substantially lower burden on patients which is of particularly importance in pediatric patients.

By measuring these transit times in both the terminal ileum and the sigmoid colon, we were able to evaluate the functionality of specific intestinal segments, even in the absence of morphological changes, and localize dysfunction to either the small intestine or colon, thereby allowing separate assessment of small and large bowel transit. Another observation was that patients with symptoms during the examination had a faster intestinal transit, reflecting increased gut motility.

Furthermore, we defined a cut-off point, which enables a more rapid and straightforward interpretation of the measurement results, to integrate this new diagnostic into clinical care. This facilitates real-time protocol adaptation and allows for comparability both between patients and, over time, within the same individual.

As non-specific gastrointestinal complaints are common in pediatric care^27,28^, CE-MSOT method holds promise for future diagnostic use to enable objective evaluation of previously unclear symptoms through functional imaging of the gastrointestinal tract. Patients with unexplained gastrointestinal symptoms are often stigmatized, even by physicians and nursing staff^29^, because of the non-objectifiable nature of these disorders^30^. CE-MSOT might reduce stigmatization in the future, while simultaneously promoting a deeper understanding of symptom pathophysiology among physicians.

Several limitations of this study should be acknowledged. For instance, this study was conducted with only a small and heterogeneous patient cohort and only three patients had a positive lactose breath test. Also, the observation periods were relatively short with a maximum of 163 min. As a result, no signal could be detected in the ileum of one patient, and no signal in the sigmoid colon of four patients within the measured timeframes. Another limitation is that measurements were taken only at two sites of the gastrointestinal tract, the terminal ileum and the sigmoid colon. Also, it cannot be ruled out that the fasting state combined with liquid bolus administration of ICG may modify the osmotic effects of lactose, potentially leading to accelerated gastrointestinal transit. A comparison to existing transit assessment methods (e.g., scintigraphy, smart pills and MRI) would be preferable, however, difficult to assess due to ethical reasoning in pediatrics.

This pilot study demonstrates the feasibility, safety and potential of CE-MSOT for assessing intestinal transit time in children. By establishing a cut-off value, this method can be integrated into clinical workflows and future research. Gastrointestinal transit times may serve as a biomarker of digestive function - for example, as a diagnostic tool for patients with unexplained gastrointestinal symptoms or functional abdominal pain, or to localize functional disturbances to specific intestinal segments. Our approach might help in objectifying previously unclear symptoms with no anatomical correlation and thereby offer better understanding in underlying causes for patients and physicians. However, future double-blind studies with larger cohorts are needed to assess diagnostic accuracy of this innovative method.

## Methods

### Ethics statement

The study was approved by the local ethics committee of the University Hospital Erlangen (24-280-Bm), registered (NCT06617364) and conducted in accordance with the Declaration of Helsinki. Also, the study was registered in a publicly accessible primary register that participates in the WHO International Clinical Trial Registry Platform: ClinicalTrials.gov, TRN: NCT06617364, Registration date: 24 September 2024. All study participants as well as their legal guardians provided written informed consent after a thorough consultation with a physician.

### Design and flow of the study

Between October 2024 and February 2025, patients who consulted the Department of Gastroenterology of the Department of Pediatrics and Adolescent Medicine of the Friedrich-Alexander-University Erlangen-Nuremberg for gastrointestinal complaints that required H2 breath test were offered participation in the study. The patients presented with a variety of intestinal complaints, most notably abdominal pain (Table 2).

**Table 2:** Overview of the gastrointestinal transit time measured using CE-MSOT^c^

patient	ICG^a^ in terminal ileum [min]	ICG in sigmoid colon [min]	abnormal stool consistency	H_2_-breath-test-positive	abdominal pain^b^	feeling of fullness^b^	bloating^b^	defaecation^b^
01	146	>146^d^	diarrhea, obstipation	-	-	-	-	-
02	124	>150^d^	obstipation	-	-	-	-	-
03	84	84	obstipation	-	yes	yes	-	yes
04	41	41	diarrhea	-	-	-	-	-
05	76	>133^d^	obstipation	-	-	-	-	-
06	60	85	-	-	yes	yes	yes	yes
07	13	127	obstipation	yes	-	-	-	-
08	>123^d^	>123^d^	diarrhea, obstipation	yes	-	-	-	-
09	90	163	-	-	yes	-	-	-
10	64	103	obstipation	yes	yes	-	-	yes

In patient with no ICG signal during the observed period, the last imaging was used as time value.

^a^Indocyanine Green.

^b^Abdominal pain, feeling of fullness, bloating and defecation were shown during the examination.

^c^Contrast-enhanced Multispectral optoacoustic tomography.

^d^Not detectable during the examination period.

In these cases, an H2 breath test was performed to rule out lactose intolerance. This allowed us to integrate the novel measurement method into an already well-established clinical workflow and to assess its practical applicability under real-world clinical conditions. General exclusion criteria were pregnancy, breastfeeding mothers, tattoos in the area of the examination and subcutaneous fat tissue over 3 cm. With regard to ICG, known hypersensitivity to ICG, sodium iodide or iodine, hyperthyroidism, focal or diffuse thyroid autonomy or treatment close in time to check thyroid function with ingestion of radioactive iodine within two weeks or after the study were exclusion criteria. Impaired kidney function or the use of the following medications also led to exclusion: Beta-blockers, anticonvulsants, cyclopropane, bisulphite compounds, haloperidol, heroin, meperidine, metamizole, methadone, morphine, nitrofurantoin, opium alkaloids, phenobarbital, phenylbutazone, probenecid, rifampicin, any injection containing sodium bisulphite.

### Data acquisition

Before the day of the examination, all subjects fasted from 10 p.m. the previous day until the start of the examination at 8 a.m. All study participants underwent hybrid MSOT/Reflectance ultrasound computed tomography of the intestine as a baseline measurement. Subsequently, all participants received either 12.5 mg (weight <30 kg) or 25 mg (weight ≥30 kg) ICG depending on their weight diluted in 500 ml of lactose-enriched water. Immediately after ingestion, a further measurement was taken, followed by at least four additional intestinal measurements using MSOT, each 20 to 30 min apart (Fig. 1). The individual measurements of the H2 breath test were also performed at intervals of 20 to 30 min, allowing the intestinal transit time assessment to be carried out during the breaks in between, without resulting in any additional time burden for the participants. Anatomical locations for imaging were terminal ileum and sigmoid colon (Fig. 1). To ensure that the same location was recorded in each measurement, the examiner used the anatomical landmarks of the terminal ileum and the sigma as orientation. Also, the examiner stayed the same across nearly all measurements.

### MSOT device

Multi-spectral optoacoustic tomography (MSOT) was performed using an Acuity ECHO CE system (iThera Medical GmbH, Munich, Germany) to visualize intestinal segments. This system integrates ultrasound (B-mode) with optoacoustic imaging to enhance anatomical localization (Fig. 1)^31^. A handheld 2D detector, combined with transparent ultrasound gel (Aquasonic Clear, MDSS GmbH, Hannover, Germany), was used to capture a minimum of three images per location. During imaging, both subjects and all treating people wore laser safety goggles. Anatomical regions (terminal ileum and sigmoid colon) were identified using B-mode imaging, while optoacoustic signals were recorded at wavelengths of 700, 730, 760, 800, 850 and 900 nm.

Regions of interest (ROIs) were defined within the intestinal lumen directly after image acquisition using B-mode guidance (Fig. 1) and verified by a certified ultrasound examiner to ensure consistency across scans. The presence of stool in the bowel lumen served as an anatomical landmark. The analysis was performed using the iLabs software (V 1.3.25, iThera Medical GmbH, Munich, Germany). To quantify individual wavelengths and unmixed ICG signal levels (derived from wavelengths 700, 730, 760, 800, 850 and 900 nm), the mean of the highest 10% of pixels within each ROI was used in batch mode analyses. Linear spectral unmixing for ICG was performed using a 6.5 µM plasma reference spectrum, based on prior studies^16^.

### Statistics

The GraphPad Prism software (Version 10.2, GraphPad Software Inc., San Diego, USA) was employed for statistical evaluation. The descriptive data was summarized as numbers and percentages, as mean and standard deviation (SD) or as median and interquartile range (IQR). A normal distribution was not assumed. ICG data were assed as both absolute value and after normalization to baseline. The presence of the oral administered ICG within the intestinal lumen was visually detected in real-time during the measurements (Fig. 1). Additionally, after defining the region of interest (ROI), the corresponding spectral data can be displayed, showing a characteristic ICG-specific peak at around 800 nm^32^. For a quantitative evaluation a cut-off point for the ICG signal was established and set at twice the baseline value across all measurements. This resulted in a cut-off value of 0.001 arbitrary units. (Fig. 1, Fig. 2). Groups were then compared by quantifying the unmixed ICG signal. In addition to comparing the quantified values with the predefined cut-off thresholds, we visually assessed the signal in each patient and compared the measured spectrum with the reference spectrum to determine whether the signal represented true ICG absorbance or a potential artifact.

## Data Availability

The datasets generated and/or analyzed during the current study are not publicly available due to protections of patients privacy but are available from the corresponding author on reasonable request.
