# Author Correction: Non-invasive measurement of accelerated gastrointestinal transit in pediatric patients using Contrast-enhanced Multispectral optoacoustic tomography

**DOI:** 10.1038/s44303-026-00177-4

**Published:** 2026-09-07

**Authors:** Luisa Caselitz, Merle Claßen, Adrian Buehler, Lars-Philip Paulus, Henriette Grieshaber-Bouyer Mandelbaum, Emmanuel Nedoschill, Joachim Wölfle, André Hoerning, Ferdinand Knieling, Adrian Philip Regensburger, Felix Wachter

**Affiliations:** Department of Pediatrics and Adolescent Medicine, University Hospital Erlangen, Erlangen, Germany

**Keywords:** Gastroenterology, Medical research

Correction to: *npj Imaging* https://doi.org/10.1038/s44303-026-00169-4, published online 04 May 2026

“In this article the author name “Adrian Buehler” and André Hoerning” was incorrectly written as “AdrianBühler and André Hörning”. The original article has been corrected.”

